# Socio-Economic Disparities in Pediatric Viral Gastroenteritis: A Comparative Study of Clinical Outcomes and Management in Hospitalized Children with Rotavirus, Adenovirus, and Norovirus Infections

**DOI:** 10.3390/children12070856

**Published:** 2025-06-27

**Authors:** Ioana Arbanas, Gabriela Coja, Alice Bilotta, Raluca-Ileana Lixandru, Oana Patran, Laura Bleotu, Oana Falup-Pecurariu

**Affiliations:** 1 Department of Medical and Surgical Specialties, Faculty of Medicine, Transilvania University of Brașov, 500036 Brașov, Romania; 2 Children’s Clinical Emergency Hospital of Brasov, 500063 Brașov, Romania

**Keywords:** rotavirus, vaccination, Romania, poor social backgrounds

## Abstract

Background: Acute viral gastroenteritis (AVG) still has an impact on children under 5 years old in developing countries. In Romania, vaccination against Rotavirus is not included in the National Immunization Program. Children from poor social backgrounds represent 48% of the patients hospitalized in Children’s Clinical Hospital of Brasov. The use of antibiotics in Romania is high. Methods: The retrospective study enrolled 1054 children, 0–48 months of age, hospitalized in the Emergency Clinical Hospital for Children Brasov between January 2022–December 2023, for Rotavirus, Adenovirus or Norovirus acute gastroenteritis. Children presenting nosocomial infections were excluded. All children that have met the previously mentioned criteria, presenting positive stool samples (immunochromatography method) for Rotavirus, Adenovirus or Norovirus were included in the study. Results: Out of 1054 cases of AVG 782 (74.2%) were due to Rotavirus, 196 (18.5%) to Adenovirus and 76 (7.3%) to Norovirus. A total of 477 (45.3%) patients came from poor social backgrounds and 577 (54.7%) children from good social backgrounds. Rotavirus infection was equally prevalent in both groups (*p* < 0.35). Children from poor social backgrounds presented an average age of 11 months (SD ± 9; range 14 days–48 months) and a hospitalization average of 5.86 days (SD ± 3.67; range 1–22 days) compared to the others, who presented an average of 21 months (SD ± 15; range 26 days–48 months) and hospitalization average of 5.20 days (SD ± 2.51; range 1–18 days) *(p* < 0.01; *p* < 0.01). Severe dehydration presented 267 children from poor settings (56%) and 186 (17.6%) received antibiotics, compared to the other group—224 children (38.8%) with severe dehydration and 216 (20.4%) receiving antibiotics. The most used antibiotic for both groups was Ceftriaxone (53.9% and 57.6% of all AVG). Conclusions: Rotavirus is the leading AVG cause. Children from poor backgrounds were younger, more dehydrated, hospitalized longer.

## 1. Introduction

Viral gastroenteritis is a common health issue in children younger than 5 years old [1]. Worldwide, 68% of diarrheal disease occurs in young children [2]. In countries where the rotavirus vaccination has not been included in the National Immunization Program, rotavirus remains the most common viral cause of acute viral gastroenteritis (AVG) [3]. In Europe, rotavirus gastroenteritis accounts for between 75,000 and 150,000 hospitalizations and up to 600,000 medical visits annually [4]. Due to its widespread prevalence, rotavirus and its effects on the human body have been extensively studied. However, less attention has been given to potential differences in the frequency of rotavirus infection among specific population subgroups, as well as the role socio-demographic factors may play in the severity and treatment of viral gastroenteritis. Despite the availability of rotavirus vaccines since 2006, rotavirus is still estimated to cause approximately 200,000 deaths annually among children under five years old worldwide [5]. Several factors contribute to the higher vulnerability of children to this infection, including immature immune systems and increased exposure to high-risk environments [6]. While prevalence of rotavirus infection among hospitalized children with diarrhea is relatively consistent worldwide (around 30–50%), over 90% of deaths from rotavirus occur in low-income countries [7,8]. This highlights that the severity of the infection is often linked not only to the virus itself but also to complications such as dehydration, which can be life-threatening in children with underlying conditions like malnutrition or co-infections. Children living in developing countries experience a higher incidence of severe illness and comorbidities [9]. The Roma population, a culturally distinct community dispersed throughout various regions of Europe, often experiences socio-economic deprivation and limited access to healthcare services, including primary care [10].

Although rotavirus-related deaths may seem rare in some regions, evidence from Romania between 1998 and 2003 showed an incidence of fatal rotavirus gastroenteritis comparable to that seen in low-income countries [11]. This emphasizes the importance of closely monitoring the distribution and impact of the virus, especially among vulnerable populations, such as children from disadvantaged socio-economic backgrounds.

This study aims to examine how acute viral gastroenteritis (AVG) differs in severity and treatment among children under two years from two distinct socio-economic backgrounds. The study population includes children from predominantly Romanian households as well as Romani households. In Romania, the rotavirus vaccination is not part of the National Immunization Program, even though rotavirus is the leading cause of viral acute gastroenteritis. In general, acute gastroenteritis resolves on its own and does not require antibiotic treatment. However, inappropriate use of antibiotics can lead to antibiotic-associated diarrhea, other complications, and long-term antibiotic resistance. Romania has the highest rate of community antibiotic consumption in Europe [12], along with some of the highest levels of antimicrobial resistance [13]. Children from low-income families are less able to prevent antibiotic-resistant infections due to limited access to clean drinking water, proper sanitation, hygiene, and healthcare. When combined with poor nutrition and substandard housing, these conditions significantly increase the risk of infectious disease in disadvantaged families and communities [14]. According to the World Health Organization, more than 8.4 million people died from infectious diseases in 2016, with most of these deaths occurring in low- and middle-income countries among economically disadvantaged populations [14]. Additionally, children from poor backgrounds are often prescribed antibiotics more frequently, due to a variety of factors [15]. The aim of the study is to assess the clinical characteristics, severity, treatment patterns, and comorbidities from good and poor socio-economic backgrounds in children under 48 months hospitalized for rotavirus, adenovirus or norovirus gastroenteritis.

## 2. Materials and Methods

### 2.1. Study Design

**Scheme 1:**
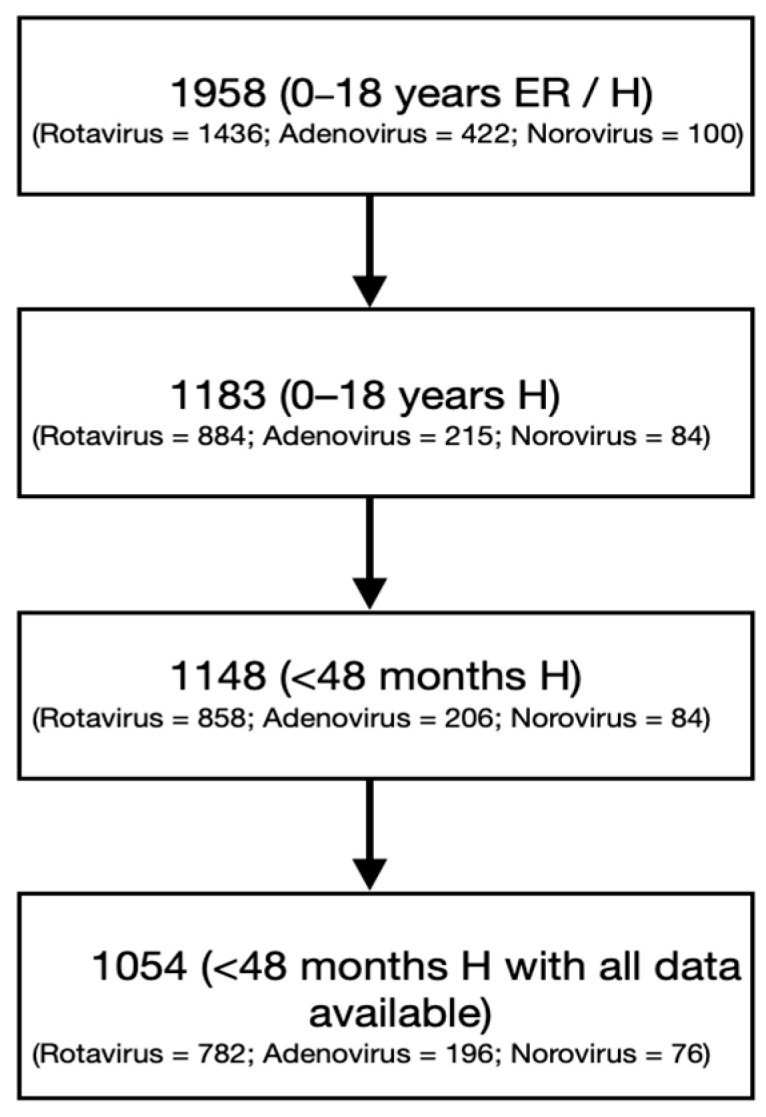
Selection flow of pediatric gastroenteritis cases with complete data (rotavirus, adenovirus, norovirus). ER = Emergency Room; H = hospitalized.

During the years 2022 and 2023, a total of 15,749 tests for rotavirus, adenovirus, and norovirus were conducted at our hospital in children aged 0–18 years who presented to the Emergency Room, whether they were subsequently discharged or hospitalized. Specifically, 7244 tests were performed for rotavirus, 7258 for adenovirus, and 1247 for norovirus. From these tests we selected the positive cases as follows (Scheme 1).

This study is an observational, retrospective, and descriptive study that enrolled hospitalized children 0–48 months reported during January 2022–December 2023 by the microbiology laboratory of the Children’s Clinical Hospital of Brasov with a positive stool specimen for rotavirus, adenovirus or norovirus. Children with nosocomial infections were excluded from the study, as well as children that did not have all available data included in their electronic archived data.

Children’s Clinical Hospital of Brașov has an average of 220 beds and an average of 9000 admissions per year.

### 2.2. Laboratory Diagnostics

The hospital laboratory used rapid diagnosis tests by immunochromatography method. The professional in vitro diagnostic tests that were used for the detection of rotavirus and adenovirus—the Rotavirus and Adenovirus Combo Rapid Test Cassette from Tody Laboratories with a relative sensitivity of 97.3% and a relative specificity of 97.1% (Bucharest, Romania) [16]. For the detection of norovirus in stool samples was used Nadal Norovirus GI/GII test (cassette test) which has a sensitivity of 97.9% (Moers, Germany) [17].

Socio-demographic data, clinical symptoms, and treatment details were collected from electronic medical records and patient files archived at the hospital.

### 2.3. Ethics

All ethical standards concerning personal data protection were strictly observed, and no information included in the study can be used to identify any child. Upon admission, all caregivers signed informed consent forms for hospitalization and the potential inclusion of data in retrospective studies. The study was approved by the Ethics Committee of the Clinical Children’s Hospital Brasov (Approval No. 9/4905/18.03.2025).

### 2.4. Definitions

We defined viral acute gastroenteritis as 3 or more stools per day caused by a known viral pathogen. This threshold of 3 or more stools per day is not stated verbatim in guidelines, the definition aligns with the clinical and laboratory criteria described by the Infectious Diseases Society of America and the American Society for Microbiology [18].

Children from poor socio-economic backgrounds were defined as those living in families with limited access to healthcare professionals (such as family doctors), presenting with poor hygiene upon admission, using well water as their primary water source, lacking electricity, or living in overcrowded conditions (more than four family members in a single room).

Children from good socio-economic backgrounds were defined as those living in families with easy access to healthcare professionals (such as family doctors), presenting with good hygiene at admission, using safe water sources, having access to electricity, and living in households with fewer family members per room.

For defining the severity score of dehydration, we used the World Health Organization Clinical Dehydration Scale at admission [19]. The clinical parameters that were used were: general appearance, eyes, thirst, and skin turgor.

For the Vesikari score, we used the following parameters: for each of the following parameters, one to three points were given: the maximum number of stools/day; diarrhea duration (days); the maximum number of vomiting episodes/day; vomiting duration (days); dehydration, defined as weight loss percentage and treatment (rehydration/hospitalization); and temperature at admission (Celsius degrees) [20].

Bacterial coinfections: children that presented a value of C reative protein > 1 mg/dL associated with leucocytosis (leucocytes > 15.000/µL and fever (>38 degrees Celsius).

### 2.5. Statistic Analysis

To evaluate the associations between categorical variables such as etiology, age distribution, distribution of comorbidities, and treatment across socio-economic groups, we employed the Chi-square test of independence. Fisher’s Exact Test was applied when expected cell counts were less than 5. For continuous variables, a two-sample *t*-test was conducted to compare the means between the two groups. The resulting *p*-values were used to determine the statistical significance of the observed differences, with a significance level set at *p* < 0.05.

Analyses of the study were carried out using Microsoft Excel Office 365 and MATLAB 2021.

## 3. Results

### 3.1. Study Population Characteristics

We enrolled 1054 children. A total of 477 (45.3% of all AVG) patients came from poor social backgrounds and 577 (54.7% of all AVG) children from good social backgrounds. The viral etiology for acute gastroenteritis is presented in Table 1. Rotavirus was the most frequently identified virus across both socio-economic groups (χ^2^ = 8.997, *p* = 0.011).

For the poor social background children group, the most frequent age group was 1–12 months (24.8% out of all AVG) compared to children coming from a good social background who were more frequently above 12 months age—419 children (39.7% out of all AVG), Table 2.

Children from poor socio-economic backgrounds presented at 11 months (SD ± 9; range 14 days–48 months) compared to the other studied group, which presented an average of 21 months (SD ± 15; range 26 days–48 months) (*p* < 0.01).

The hospitalization average days was 5.20 days (SD ± 2.51; range 1–18 days) for children coming from good socio-economic backgrounds and 5.86 days (SD ± 3.67; range 1–22 days) for children coming from poor socio-economic backgrounds (*p* < 0.01).

Comorbidities were observed in 155 children (26.8%) from the good socio-economic group and in 160 children (33.5%) from the poor socio-economic group. (Table 3).

20 (1.89% of all AVG) children from good socio-economic backgrounds were vaccinated against rotavirus disease and 1 (0.09% of all AVG) child from the poor socio-economic background.

### 3.2. Treatment and Severity

A total of 347 children (32.9% of all AVG) from higher socio-economic backgrounds were hospitalized for observation, administration of intravenous fluids, and symptomatic management, whereas 268 children (25.4% of all AVG) from lower socio-economic backgrounds received the same treatment during their hospital stay, Table 4.

Within the good socio-economic background, 216 children received antibiotics compared to 186 children coming from the poor socio-economic background. A total of 43 children (89.5%) of neonates and young infants (less than 3 months) received antibiotics in the group coming from good socio-economic backgrounds and 90 children (100%) of neonates and young infants (less than 3 months) received antibiotics in the group coming from poor socio-economic backgrounds. The most used antibiotic for both studied groups was Ceftriaxone 53.9% (122 children) for children with good socio-economic backgrounds and 57.6% (106 children) for children with poor socio-economic backgrounds followed by Ampicillin in combination with Gentamicin 31.5% (58 children) for children coming from poor socio-economic backgrounds and 27.8% (63 children) of children received Ampicillin for children from good socio-economic backgrounds.

Bacterial coinfections were observed in 188 children (32.5%) from the good socio-economic group and 105 children (22%) from the poor socio-economic group. Bacterial coinfections were as follows: acute otitis media (78 cases from the good socio-economic group vs. 15 cases from the poor socio-economic group), undetected source bacterial infections (67 cases vs. 26 cases), respiratory tract infections (11 cases vs. 54 cases), and urinary tract infections (32 cases vs. 10 cases).

**Figure 1:**
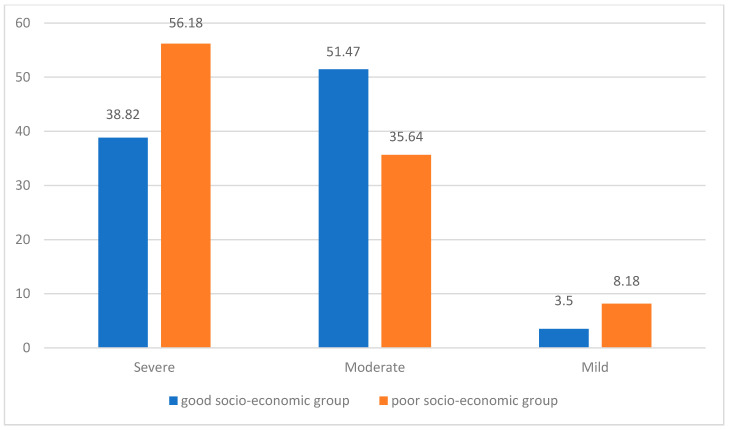
Dehydration severity in relation to socio-economic group.

Severe dehydration according to the WHO Dehydration Scale was present in 267 children (56%) from a poor socio-economic background and 224 children (38.8%) of children from a good socio-economic background) children (Figure 1).

Rotavirus gastroenteritis presented the highest Vesikari score in both studied groups (11.6 vs. 11.8) with a *p* value < 0.01 for both comparison with adenovirus and norovirus. Children coming from poor socio-economic backgrounds did not present a statistically higher Vesikari score compared to children coming from good socio-economic backgrounds, Table 5.

## 4. Discussion

Acute gastroenteritis is very common in children, and most cases are caused by viral agents [21]. The most common viruses responsible are rotavirus, norovirus, and adenovirus [21]. Today, rotavirus remains the most common cause of both diarrhea and diarrhea-related deaths [22]. The burden of viral gastroenteritis is most significant in developing countries, where the majority of deaths occur, primarily due to rotavirus [23]. In our study, rotavirus was the most frequently detected virus, followed by adenovirus and norovirus in both studied groups. At the same hospital in Brasov, Romania, a survey conducted between 2015 and 2021 also showed a high incidence of rotavirus [24]. In Romania, rotavirus vaccination is not mandatory but is available for purchase on the private market [24]. According to a 2023 report by the National Institute of Statistics (INS), nearly one in five people (19.8%) in Romania experience severe material and social deprivation—“21.1% of the resident population lived in a household whose income was lower than the threshold set at the level of 60% of the median available income per adult equivalent” [25].

In our study, in the group of children from poor socio-economic backgrounds, the most common age group was 1–12 months, in contrast to children from better socio-economic backgrounds, where the majority were over 12 months old. These findings are consistent with the previous research showing that children under two years of age in developing countries are more frequently affected by such illnesses [26]. The average age of children from poor socio-economic was lower—11 months compared to 21 months in the other group— and the difference was statistically significant. The younger age of children from poor socio-economic backgrounds may reflect multiple interrelated factors, such as limited access to preventive healthcare, lower vaccination coverage, delayed recognition of early symptoms by caregivers, and overall increased vulnerability to infections in infancy due to suboptimal living conditions. Infants in disadvantaged settings may also be exposed to pathogens earlier due to crowded households or inadequate sanitation. There is a need for further research to explore these socio-environmental determinants in greater depth.

Hospitalization is longer for children from disadvantaged backgrounds as is shown in our study. Children coming from poor socio-economic backgrounds presented longer hospitalization days—5.86 days compared to the other group who presented an average of 5.21 days (*p* < 0.01). Children who were hospitalized for more than seven days were those with chronic comorbidities.

Viral acute gastroenteritis is generally considered to have a simple, effective, and low-cost treatment approach, which includes rehydration, continued oral feeding, and symptom-relieving medications [23]. Oral rehydration solutions and zinc are particularly important, as studies have shown they can reduce the duration of hospitalization and improve gastrointestinal symptoms [23]. In our study, none of the children received zinc, as it is not included in our hospital’s treatment protocol. Most children in both studied groups received intravenous rehydration along with intravenous electrolytes and salts. All children continued oral feeding and were treated with symptom-relief medication, such as diosmectite and simethicone, in accordance with hospital protocol.

Antibiotic use is very high in Romania, which has the highest rate of community antibiotic consumption in Europe [27] and one of the highest levels of antimicrobial resistance on the continent [13]. In our study, a percentage of 21.4% and 17.4% of children with a higher rate in children coming from good socio-economic backgrounds received additional antibiotic treatment. This approach appeared to be primarily associated with the very young age of the patients (under 3 months). A recent study conducted in Israel found that 18.1% of children with viral gastroenteritis received unnecessary antibiotic treatment [28]. The study also showed an inverse association between rotavirus vaccination and the likelihood of receiving unnecessary antibiotics [28]. Although studies in the literature show that children from disadvantaged backgrounds are prescribed antibiotics more frequently than those from better socio-economic backgrounds [29,30], our findings show that children from good socio-economic backgrounds received 4% more antibiotics than children from poor socio-economic backgrounds. This might be related to the acute bacterial coinfection that children presented and better monitorization of acute and chronic pathologies in children from better socio-economic backgrounds because of their easier access to healthcare system.

Using the WHO Clinical Dehydration Scale, a statistically significant difference in dehydration levels was observed between children from poor and good socio-economic backgrounds. Children from disadvantaged backgrounds experienced more severe dehydration, likely due to limited access to pharmacies, healthcare professionals, and hospitals [31].

Regarding the Vesikari score, rotavirus infections were the most severe among both study groups compared to infections caused by adenovirus and norovirus. No statistically significant difference was observed in children from poor socio-economic backgrounds compared to those from better socio-economic backgrounds regarding severity for all three of the AVG viruses studied. In the Netherlands—a high-income country that has not implemented rotavirus vaccination in its National Vaccination Schedule—several studies have shown that rotavirus infections are more severe than infections caused by other enteric viruses [32,33].

The introduction of rotavirus vaccination has demonstrated clear and substantial public health benefits, particularly in countries geographically close to Romania. In the Republic of Moldova, the inclusion of the rotavirus vaccine in 2012 led to a dramatic reduction in hospitalizations among children under five, with rotavirus-positive admissions decreasing from 45% before the vaccine to 25% and 14% in the first and second years after its introduction, respectively [34]. The vaccine showed 79% effectiveness against rotavirus-related hospitalizations and also conferred indirect protection to unvaccinated cohorts, highlighting the effects of herd immunity [35]. Broader European data reinforce these findings: systematic reviews covering Central and Eastern Europe report vaccine effectiveness ranging from 68% to 98% with reductions in rotavirus hospitalizations between 65% and 84% [35]. Across European healthcare settings, significant declines in disease burden, hospitalizations, outpatient visits, and healthcare costs have been consistently observed, especially among children under five [35].

Several limitations should be considered when interpreting the findings of this study. First, the sample size, while sufficient for general trends, may be inadequate to detect smaller but clinically relevant differences between viral pathogens across socio-economic groups. Second, there is potential for selection bias, as children who presented to the hospital may not represent all cases of viral gastroenteritis in the community—particularly in underserved or rural areas where access to healthcare may be limited. Third, the generalizability of the results may be constrained by the study’s single-center design, which may not reflect broader national patterns of viral gastroenteritis. Another limitation would be the reliance on stool antigen testing—which may lead to underdiagnosis. These limitations underscore the need for cautious interpretation and further research using larger, more diverse populations and longitudinal data.

## 5. Conclusions

This study reinforces the idea that rotavirus remains the leading cause of acute viral gastroenteritis, with a higher severity of illness compared to other viral pathogens. The disproportionately severe impact on children from disadvantaged backgrounds—reflected in younger age at presentation, greater dehydration severity, and longer hospital stays—highlights underlying health inequities. Despite these disparities, treatment approaches did not differ between groups, and the high rate of antibiotic use across the board raises concerns about potential overuse in viral illnesses. These findings underscore the need for strengthened public health strategies, including enhanced rotavirus vaccination coverage and targeted education efforts, particularly in vulnerable populations. Future research and policy should focus on closing these health gaps and optimizing clinical management to reduce unnecessary antibiotic exposure.

## Figures and Tables

**Table 1:** Etiology of acute viral gastroenteritis in relation to socio-economic background.

	Good Socio-Economic Backgrounds		Poor Socio-Economic Backgrounds		Total	*p* Value (Chi-Square)
Virus	No. Cases	% Within Group	No. Cases	% Within Group	No. Cases	
Rotavirus	421	72.9	361	75.7	782	0.351
Adenovirus	102	17.7	94	19.7	196	0.445
Norovirus	54	9.4	22	4.60	76	0.004
Total	577		477		1054	

**Table 2:** Age distribution of study patients by socio-economic background.

	Good Socio-Economic Backgrounds		Poor Socio-Economic Backgrounds		Total	*p* Value (FET; Chi-Squared)
Age (Months)	No. Cases	% Within Group	No. Cases	% Within Group	No. Cases	
0–1	24	4.2	37	7.70	61	0.119
1–12	134	23.2	262	55	396	<0.001
≥12	419	72.6	178	37.3	597	<0.001
Total	577		477		1054	

**Table 3:** Distribution of comorbidities among children by socio-economic status.

Comorbidities	Good Socio-Economic Backgrounds		Poor Socio-Economic Backgrounds		*p*-Value (Chi-Square)
	No. Cases	% Within Group	No. Cases	% Within Group	
Allergies	21	3.63	6	1.25	0.025
Anemia	61	10.5	73	15.3	0.027
Malnutrition	12	2.07	57	11.9	<0.001
Other (Renal/cardiac congenital malformation, neurological disorders, genetic disorders)	61	10.5	31	6.49	0.026

**Table 4:** Treatment management of patients by socio-economic background.

Treatment	Good Socio-Economic Backgrounds		Poor Socio-Economic Backgrounds		Total	*p*-Value
	No. Cases	% Within Group	No. Cases	% Within Group	No. Cases	
1	1	0.18	0	0	1	1.0
2	3	0.52	25	5.20	28	0.001
3	347	60.2	268	56.2	615	0.307
4	226	39.1	184	38.6	410	0.951

1 = admitted for observation only; 2 = admitted for observation and intravenous fluids; 3 = admitted for observation, intravenous fluids, and symptomatic treatment; 4 = admitted for observation, intravenous fluids, symptomatic treatment, and antibiotics.

**Table 5:** Average Vesikari scores for stool pathogens according to socio-economic background.

Average Vesikari Score						
Virus	Adenovirus		Rotavirus		Norovirus	
	Good socio-economic backgrounds	Poor socio-economic backgrounds	Good socio-economic backgrounds	Poor socio-economic backgrounds	Good socio-economic backgrounds	Poor socio-economic backgrounds
	11.2	11	11.6	11.8	11.1	10.5
*p* value (T-test)	0.13		0.28		0.10	

## Data Availability

All data is available in the study.

## Abbreviations

The following abbreviations are used in this manuscript:

AVG	Acute Viral Gastroenteritis
